# Johns Hopkins Hospital

**Published:** 1914-07-25

**Authors:** 


### Johns Hopkins Hospital.

A signal compliment has been paid to Dr. Thomas Lewis, editor of *Heart*, and assistant physician to University College Hospital, by his selection to deliver the Hester Lectures in October next at the Johns Hopkins Hospital School, Baltimore. The occasion is one of more than ordinary importance, since it marks the twenty-fifth anniversary of the opening of the Johns Hopkins Hospital and the twenty-first anniversary of the opening of the medical department in connection with it. Dr. Lewis, it is also noteworthy, is the first clinical investigator who has filled the position of Hester Lecturer. The titles of his three lectures are not yet announced, but they are certain to deal with cardiology, and probably with some of those problems which his own researches have done so much to elucidate.

